# Medical, Pharmaceutical and Dental Directory

**Published:** 1895-02

**Authors:** 


MEDICAL PHARMACEUTICAL AND DENTAL DIRECTORY.—Geo. Keil, publisher and editor, Philadelphia.

This Directory is for the States of Pennsylvania, New York, New Jersey, Maryland, Delaware and District of Columbia; and for these States it is very complete, and will be of much value to those within them, and to a goodly number outside. It also contains much valuable information, in addition to the list of names of these three departments. It can be obtained through any dental depot.

